# Remedies for the Neglect of Science

**Published:** 1916-06-10

**Authors:** 


The Hospital

The Workers' Newspaper of Administrative Medicine and Institutional Life, Administration, National Insurance and Health.

No. 1565, Vol. LX. SATURDAY, JUNE 10, 1916. PRICE ONE PENNY.

### REMEDIES FOR THE NEGLECT OF SCIENCE.

The Memorial presented by the Professors of the Imperial College of Science and Technology to Lord Crewe is a document very appropriate to the day and hour. In a rough school the nations of the world are learning that the application of scientific knowledge governs important issues. Even questions apparently remote from the laboratory—such, for example, as national freedom and public law—wait for an answer on its decision. And there is general agreement that when the industrial era returns, success in the inevitable international competition will go to the peoples whose activities are regulated by scientific truths. What was at one time largely regarded as an enterprise having little association with practical life is gradually becoming recognised as an essential condition of national existence. Science is concerned, to put the matter briefly, with the truth about the world in which we live, and only peoples who know this truth and accommodate themselves to its demand can struggle successfully for a place in the sun. All this just now is receiving emphatic demonstration. The anxious question is to find the method which will fasten the lesson on the public consciousness so securely that practical application of it may be achieved. There have been in the past many announcements of these truths both by individuals and by committees, but the teaching has reached neither the mass of the people nor the conscience of our governing classes. The experiences of the present and the anticipations of the future make the question prominent to all eyes. It remains to be seen whether leadership and public opinion will be equal to the opportunity.

One great virtue of the Professors' Memorial is that it deals not with generalities but with specific proposals. It takes for granted the proposition that the national life needs for its force and success more guidance by scientific knowledge, and it wisely recognises that to attain this end the status of scientific work and training must be raised. In the convictions of those who are set in authority there must be established the clear certainty that—in its method, its outlook, its discipline—science is worthy of the attention of the best minds, and this, whether it is regarded as an educational influence or as a utilitarian necessity. To try to attain this end the Professors call upon the Government through some of its leading members to speak plainly to the country on the question of national education, concerning which there is so much "lethargy, misconception, and ignorance." Probably everyone will agree that before matters are put on a satisfactory basis the Government will have actively to intervene. Yet it might be wished that some of the men of science themselves could discover that they possessed the capacity to conduct an educational campaign. One of the weaknesses of the position is that to so many the pursuit of science seems to shut off its most successful followers from the obvious currents of national life and affairs, and either to hide them in the unknown or to label them as cranks—clever, no doubt, but eccentric. It is not in the least an exaggeration to say that even among the reputed educated classes a scientific worker is often regarded as a kind of superior conjurer. The best method for getting rid of this absurd notion lies with the scientific men themselves. Let some of them show that they study the conditions of success in life as well as of success in the laboratory, and that as an educated public opinion is one of these conditions, let them set themselves to create it. We cannot call forth a Huxley at will, but were such a discovery to be made, we fancy he would have a more flexible audience than any politician. In other words, we venture to say that the professors, or at least some of them, must come out of their laboratories a little more and take an active share in the educational campaign. Call upon the Government by all means, but let the learned men put their own shoulders to the wheel.

In addition to a better informed public opinion relative to scientific method, what is needed is the creation of opportunities for lads who show ability in their early school training; and especially must openings be secured for the recognition and encouragement of this ability when it shows itself in the direction of scientific studies. Under existing conditions this encouragement exists mainly in the classical and traditional departments, and even in those schools where science has some sort of a footing it suffers an inferior status and enjoys but scanty prizes. If good results are to follow, these handicaps must be removed. The teacher of science must receive pay and position equal to those of his classical colleague, and the boy with a natural bent for science must be searched for with a zeal not less than that devoted to the discovery of his orthodox fellow. From secondary schools and technical schools and evening classes avenues open to ability must lead to the wider atmosphere and larger opportunities of the universities, and these must not be blocked by hindrances created by academic prejudices or by out-of-date customs. The best brains of the rising generation are not only the wealth of the nation but also its security. They must have their chance both for the sake of the individual and in order that the nation may not perish. Discipline by sad experiences the people are ready to learn. Is there anyone able to take occasion by the hand? Of course, there lies behind all the new proposals the question of money. So long as it may be truly said that science does not pay it may have its select spirits, but hardly a body of busy workers. It is here that Government will have to take a hand. Endowments and prizes and scholarships have in the past gone largely to the encouragement of the older learning. If science is to collect its disciples it must have money for the purpose, and the charge may be defended as a national interest of the first order. Both the older learning and the new have their values. But it is time that the latter had a fairer field than it has hitherto enjoyed.

